# ‘Important, but difficult’: Swedish primary care professionals’ perceptions and experiences of dealing with violence against women: an interview study

**DOI:** 10.1186/s12875-024-02489-z

**Published:** 2024-07-16

**Authors:** Ann Öhman, Carmen Vives -Cases, Kerstin Edin

**Affiliations:** 1 Umeå Centre for Gender Studies, Umeå University, Umeå, SE-90187 Sweden; 2 Dept of Community Nursing, Preventive Medicine and Public Health and History of Science, University of Alicante, Alicante, Spain; 3 CIBER of Epidemiology and Public Health, CIBERESP, Madrid, Spain; 4 Department of Epidemiology and Global Health, Umeå University, Umeå, Sweden

**Keywords:** Violence against women, Professional competence, Knowledge, Policy, Management, Healthcare organisation, Primary care

## Abstract

**Background:**

Men’s violence against women is a global health problem causing physical, mental, sexual and reproductive ill-health. The World Health Organisation has estimated that every third woman in the world has been exposed to physical and/or sexual violence. Swedish primary care is central for victims of violence, as it is normally the first port of call for seeking healthcare. This requires professional competence on violence, and its causes. It also requires resources for working with violence prevention, disclosure and supportive actions. The aim of this study is to deepen the understanding of how primary care professionals in Sweden deal with violence against women. We analyse their viewpoints, experiences and practices of working with violence as a health problem, and especially if, and if so how, they ask patients about violence.

**Methods:**

A qualitative, explorative research design was adopted. Research interviews were conducted with 18 health professionals at eight primary care clinics. These clinics were located in four different regions, from the south to the north, in large urban areas, middle-size cities and rural areas. The interviews were voice recorded and transcribed verbatim. Thematic analysis was used to analyse the interviews.

**Results:**

Three themes, with a total of ten related sub-themes, were developed. These themes are: (a) Varying understandings and explanations of violence against women; (b) The tricky question of asking about violence; and (c) Multiple suggestions for improving primary care’s work with violence against women. The awareness of violence varied considerably, with some practitioners being highly knowledgeable and having integrated violence into their everyday practice, whereas others were less knowledgeable and had not paid much attention to violence. The very naming of violence seemed to be problematic. Several suggestions for improvements at professional, managerial and organisational levels were articulated.

**Conclusions:**

The results shed important light on the professionals’ problems and struggles when dealing with violence against women in primary care. Better support and resources from the healthcare organisation, clearer leadership and more detailed policy would improve and facilitate everyday practice. All of these factors are indispensable for primary care’s work with victims of men’s violence against women.

## Introduction

Men’s violence against women (VAW) is defined as a global public health problem causing physical, mental, sexual and reproductive ill-health [1]. The World Health Organisation (WHO) has estimated that every third woman in the world has been exposed to physical and/or sexual violence at some point during her life, in which sexual violence is often part of the physical violence [2]. In addition to the individual suffering of women, violence also incurs considerable societal costs in terms of legal, healthcare and social services expenditure. In a Swedish cross-sectional, population-based study, Öberg et al. [3] reported a 16.3% prevalence of sexual violence before the age of 18 years. Almost every second woman reported lifetime exposure to some kind of serious violence, of which 65% was sexual harassment and 20% was sexual violence. 14% of women reported violence by a former or current intimate partner (ibid.). During the pandemic years, an increase in the prevalence of intimate partner violence was observed, and the pandemic restrictions and isolation at home made it even harder for victims of violence to seek help or to escape [4–6].

In its global plan for action, the WHO highlights the importance of the healthcare sector expanding its responsibility in terms of protection against violence [2], and the Swedish government has commissioned the National Board of Health and Welfare to support and develop such work by healthcare and social services regarding men’s VAW. In line with previous research, we consider primary care a central arena for dealing with victims of violence [7], as it is normally the first port of call when seeking healthcare in Sweden. This requires professional competence on violence and its causes and consequences, including resources for working with prevention, disclosure and supportive actions. In this article, we therefore concentrate on primary care services and investigate the ways in which health professionals’ reason about how they deal with VAW in their everyday clinical practice.

A number of studies investigate health practitioners’ preparedness, attitudes and practice regarding violence against women of which many have focused on asking patients about violence exposure. For instance, Sundborg et al. [8] found that Swedish district nurses reported hesitation as to asking about violence. In line with the Swedish study, Fredriksen et al. [9] investigated Norwegian midwives experiences with screening for violence in antenatal care, and found that midwives had difficulties in asking questions about violence, especially in the beginning of a pregnancy and if they asked, they received very little information about current violence. Gutmanis et al. [10] investigated Canadian physicians and nurses and their practice of identifying patients exposed to violence. They found a number of important factors such as professional support, comfort, self-confidence and practice pressure. Practitioners’ preparedness was crucial for developing clinical routines for asking about violence. In a cross-sectional study of primary healthcare professionals in Spain, professional training on violence was identified as one of the most important factors in order to challenge hesitation and ambivalent thoughts about asking [11]. In a qualitative meta-synthesis, Hegarty et al. [12] found enhancing factors for addressing violence by health practitioners such as being committed, adopting an advocacy approach, building trust with patients, collaboration in the work team and being supported by the health system. In the Spanish primary healthcare context, Goicolea et al. [13] also identified the use of a violence-related protocol, in conjunction with accumulated experience in primary care, as the most relevant contextual conditions for a well-functioning handling of violence. They also found other positive conditions, such as an enabling team climate, having a champion social worker and having staff with training in intimate partner violence. Arboit et al. [7] studied Brazilian primary care and its potentials and limitations as to violence against women. They found similar facilitating factors as other researchers; professional experience, trust in the patient encounter, and a receptive atmosphere. Likewise, the hindering factors were similar to what others have found; denial of violence in patients and unpreparedness in the healthcare team. In addition, they also found fear among practitioners, due to presence of the perpetrators in the clinical encounter. Seymour et al. [14], indicate that educational programmes may improve healthcare professionals’ knowledge and readiness to encounter women patients exposed to violence. In sum, there is substantial agreement in the literature of the obstacles for asking about violence; being lack of professional training and knowledge, fear of asking such a sensitive question, lack of support from managers, and it not being a prioritised question at the clinic. The facilitating factors can be summarised in adequate professional training on violence and how to ask, using a protocol for asking, being committed to and building trust in the patient relationship, and an organisation that supports and prioritises tackling VAW.

In line we Boyle [15], we regard men’s VAW as existing within a broader context, with structural and societal implications. We thus embrace a feminist theoretical understanding of VAW as being societal, gendered and filled with power asymmetries [15]. Men’s violence against women is thus regarded as being rooted in unequal power relations between men and women and is considered a violation of human rights, for instance in the UN’s Sustainable Development Goals [16]. Such notions focus on gendered, societal structures as a basis for the violence, where masculine ideals of power and dominance intersect and create destructive behaviour. It is emphasised that such structures are dangerous and ‘toxic’ and constitute a threat to women’s and girls’ security [17, 18].

This study is part of a larger research project investigating the Swedish primary care sector’s work with VAW as a public health problem. Hence, in a review of official documents and governmental regulations in relation to the healthcare sector’s work with VAW, we concluded that future studies are needed at the regional level, and with healthcare professionals [19]. In addition, we found that violence was rather loosely defined in the regulations. Gender-neutral concepts of violence were frequently used in the policy documents, and the healthcare sector’s obligations were not clearly articulated, beyond the recommendation to ask questions about violence [19]. This vagueness is likely to cause confusion among healthcare professionals in their everyday practice. In the research literature on violence, concepts vary substantially [e.g. 15, 20–21]. It has been argued that concepts such as ‘domestic violence’, ‘family violence’ and ‘intimate partner violence’ (IPV) often ignore the gendered aspects of such violence and simply indicate that violence and/or conflicts exist within a family/relationship [20–22]. Such concepts are labelled ‘gender-neutral’ [19]. Due to this gender neutrality, and in accordance with our chosen theoretical framework of feminist theory on gendered violence, we use the concept VAW in this study, instead of the more commonly used IPV concept in other studies. There is a considerable knowledge gap in the research literature as to conceptualisation of violence, and we have found no articles focusing on this issue when it comes to health professionals. Hence, in the current study, we want to scrutinise the ways in which health professionals talk about VAW, what kind of concepts they use and how they understand and handle VAW. Additional previous research has focused on women’s experiences of being asked about violence in healthcare settings [23–26]. Every study emphasises the need for women exposed to violence to be listened to and to be supported by the healthcare system. Much of previous research on professionals and VAW focus specifically on district nurses and midwives. However, primary care involves a number of different professions, and due to the fact that knowledge is scarce considering primary care professions on a broader basis, we wanted to expand the scope by including other professional groups in primary care. The aim of this study was therefore to develop new knowledge on how primary care professionals in Sweden reason around VAW as a health problem. Hence, we wanted to analyse their viewpoints and understandings and explore whether they had experience of working with victims of violence, if and how they ask about violence and what their practice looks like in terms of interventions and treatments.

## Methodology

### Study context

The majority of Swedish healthcare is publicly funded (84%) and is organised into 21 regions, which have the responsibility to fund and provide healthcare for the population within that region [27]. The Swedish state governs the regions through national legislation and regulations [28], but due to strong regional self-governing, many of the decisions regarding healthcare are taken at the regional level. This implies that the handling of VAW might look rather different across various regions. VAW has been on the political agenda for decades in Sweden, resulting in improved legislation and a variety of action plans, guidelines and agendas for prevention and intervention, both in society at large, and in working life and healthcare [19]. At the time of the interviews for the current study, the national guidelines recommended healthcare professionals to ask about violence when there was a suspicion that violence may have occurred, and that all pregnant women in antenatal care should be asked, as well as all women, young people and children in psychiatric care [29, 30]. The guidelines stress the importance of providing care and support for victims of violence, as well as developing collaborations with other societal sectors, such as social security authorities and women’s shelters [31–33], and provide detailed principles about when and how to ask [29]. The guidelines have recently been revised and we come back to that in the discussion section of this article.

### Procedure, study participants and ethical considerations

A qualitative, explorative research design was adopted. In order to develop new knowledge on how primary care professionals view the issue of VAW, we decided to conduct individual research interviews. We conducted 18 on-site research interviews with health professionals at eight primary care clinics, within four regions in Sweden, from the south to the north. These primary care centres were located in large urban areas, middle-sized cities, rural areas and in high- and low-income residential areas. The sampling procedure was conducted in several steps. Prior to the visits, we contacted the managers of the primary care centres, to receive their permission to visit and conduct interviews with some of the employees. We received vast interest in our request, but due to an overload of work in primary care, some centres had to decline to participate. After receiving a positive response, both the managers and the participating professionals received written and verbal information about the project. We collected written, informed consent from all the interviewees. The interviewees were informed that participation was optional and that they could withdraw from the study at any time, without giving a reason. We also informed them that the transcripts would be held separately from lists of participants and that, when direct quotes from interviews were used, they would be anonymous with no possibility of personal identification. Lastly, they were informed that the material would be used only for research purposes. Due to the heavy work load and the time constraints that hindered some of the selected primary care centres to take part in the study, we conclude that this qualitative sample is, on the one hand a convenient sample, but on the other hand, it may also be regarded a strategic, purposive, qualitative sample with variation [34] as to professions, geographic location in the country, dense and sparsely populated areas and low-and high-income residential areas.

The 18 interviewees had various kinds of professional training: four nurses, three physicians, seven physiotherapists, three midwives and one psychologist. Some had only worked a couple of years in the profession, whereas others were almost ready to retire. All but three were women. Several had additional work tasks within their employment at the primary care clinic, such as manager, coordinator or counsellor (see Table 1).

**Table 1:** Study participants

Professions	Number	Additional work tasks
Physicians	3	Manager 1, Deputy manager 1
Physiotherapists	7	Manager 1, Rehabilitation coordinator 2, Home rehabilitation 2
Registered nurses	4	Child health & telephone counselling 1, Manager 1, Rehabilitation consultant/coordinator 2
Psychologist	1	
RN midwives	3	

Because the interview language was Swedish, they were conducted by KE and AÖ. The interviews were steered by a thematic interview guide that included the following main questions:

1. What do primary care professionals do in their everyday practice, in relation to VAW?
2. How do they label VAW? What kind of concepts do they use when they talk about violence?
3. How do staff and managers regard their knowledge, their role and their readiness to encounter and support women exposed to violence?
4. How do staff and managers view the role of primary care in relation to the care and support of women exposed to violence?
5. What do they regard as the most important actions in primary care, in order to improve care and treatment for women exposed to violence?

The interviews were held in a conversational style. We regard flexibility as a key feature of qualitative research, and therefore we probed into issues brought up by the interviewees, which were not always included in the thematic interview guide. Interviews lasted between 45 min and two hours, with a mean of approximately one hour. They were voice-recorded and thereafter transcribed verbatim.

### Analysis

We used thematic analysis in accordance with Braun and Clarke [35] to analyse the data set, which includes the entire 18 interviews. AÖ and KE started with a separate reading of the interview transcripts, coded them and made code lists for each interview. Thereafter, we compared the code lists, discussing the codes and their possible meanings. In a second step, AÖ, CVC and KE decided on themes and how to label them. In a third step, AÖ and KE re-read all the interviews to check for possible new aspects. In a fourth step, we negotiated and decided on the final themes and their related sub-themes. We conducted an inductive coding analysis [35], i.e. we did not try to fit the themes into a pre-defined coding frame. In that sense, the themes represent the ways in which the professionals talked about VAW as a health matter. Our theoretical framework of men’s VAW, based in feminist theory [15], is used in the discussion section, in order to reflect upon, and interpret, the findings. Although we made efforts to achieve variation as to professions, we did not seek to compare different professions with each other. For that, we would have needed substantially additional participants in all sub-groups. Instead, we have used a qualitative design in which we regard the interviewees as one group; namely the primary care workforce, who are expected to deal with VAW in their daily practice. Neither do we present comparisons between male and female interviewees. Only three men were interviewed, and we did not see any noticeable differences in their views, compared to those of the 15 women.

### Reflexivity

We, the three authors of this article, have been conducting research on VAW for many years, in the form of both qualitative and quantitative research. We acknowledge the view that researchers in a qualitative study, such as this, are actively engaging in the research process by our presence in the field, in the data collection procedure, in the encounters with the interviewees, and in the analytical process [36]. In accordance with Dahlgren et al. [34], and in order to better understand the interviewees’ approaches and their clinical practice, we have tried to bracket our pre-understanding during the first stages of the process; i.e. during interviewing and first reading of the transcripts. Then, during the later stages of the analytical process and in the discussion section of this article, we have utilised our theoretical understanding of VAW as being societal, gendered and filled with power asymmetries [15].

## Results

From the thematic analysis, we developed three themes with related sub-themes. They are summarised in Table 2, and are described in detail in this section. The sub-themes represent the variations in data. The main results can be summarised as “Important, but difficult”. As portrayed below, the professionals struggled with how to deal with VAW as part of their everyday clinical practice. The awareness of violence as a health matter fluctuated considerably between the interviewees, with some being highly aware and knowledgeable and having integrated issues of violence into their professional performance and practice, whereas others were less knowledgeable and had not paid much attention to violence.

**Table 2:** Themes and sub-themes developed from the thematic analysis

Theme	Sub-themes
Varying understandings and explanations of violence against women	- Types of violence - Signs of violence - Violence as conflicts within the family - Cultural and racialised expressions of violence against women - Violence as unequal gendered power relations
The tricky question of asking about violence	- Routinely asking about violence - Obstacles to asking
Multiple suggestions for improving primary care’s work with violence against women	- Guidelines, expertise and continuing education - Organisational determination - Managerial support

### Theme 1. Varying understandings and explanations of VAW

This theme is divided into five sub-themes, reflecting the variations in the data. From the interviews, it became clear that the professionals did not share similar views on what VAW can be. Instead, they talked quite differently about what violence is, i.e. how they defined violence and, accordingly, what types and signs of violence they had observed. The theme sheds light on different explanations and understandings of the root causes of VAW, why it exists and how it can be explained. These differences can partly be traced to different conceptual frameworks and theories about gender and violence, which we will consider further in the discussion section.

### Types of violence

Physical violence was the most prominent type of violence that the interviewees talked about. Less was talked about in terms of psychological violence. This was understood as another dimension of violence, usually in conjunction with physical violence. Yet others were more interested in talking about violence in close relationships, and these interviewees also seemed to be more educated on issues of VAW and how it might affect their clinical practice.

*No, I mean…violence is more than physical violence. I think it’s very important that this is made clear. That violence has a broad spectrum, so that one doesn’t forget the other parts, in healthcare, I mean, physical violence, yes, there might be many signs of such violence, but I mean, there might also be other forms of violence…that need to be handled and treated.* (Physiotherapist)

There was much less said about sexual violence. One interviewee stated that rape is not generally dealt with in primary care. Instead, he claimed that such patients should be referred to the accident and emergency department, or the gynaecological clinic, in order to meet the rape victims’ needs appropriately. The midwives seemed to be fairly aware of sexual violence, and they emphasised that sexual violence affects women, not least during pregnancy. Honour-related violence was talked about even less. The interviewees seemed to avoid the issue, and did not want to talk about the groups of people among whom this type of violence is prevalent. It seemed as though they were afraid of revealing prejudices and of being portrayed as racists. There was however also awareness among the interviewees, this is one example:

*I worked in another area before coming here, with other kinds of patients. In this area, a lot of women come from other cultures, where honour-related violence might be much more common…I experienced the education about honour-related violence that I attended as very useful.* (Physiotherapist)

#### Signs of violence

Those who were aware of the occurrence of violence, and that they might encounter patients who had been exposed to violence in their clinical practice, said that they were looking for signs such as bruises, wounds and scars; for instance, when they performed physical examinations of the patients’ bodies. In order to ask about violence, they needed clear and obvious signs that might indicate that a woman is living in a violent relationship. Then they took the time to ask and carefully examine the patient. Sometimes patients do not want to reveal the causes of bruises and other injuries, but try to explain them as due other causes, such as having fallen down the stairs or bumped into a kitchen cabinet.

*If there’s current, ongoing violence, one can see signs like bruises and scars and so on…but this is rather unusual, I would say*. (Physiotherapist)

The professionals who were more aware of violence also paid special attention to so-called ‘frequent healthcare seekers’, i.e. those who come back on a frequent basis, seeking help for multiple and varying health problems, such as stomach-ache, urinary tract problems, widespread musculoskeletal pain, headaches or anxiety. The interviewees understood that these health complaints might be a sign of something else, for instance living in a violent relationship.

*The patients often come for a long time here with me and visit me frequently. If the violence doesn’t end, they will continue coming with different health problems, and then they may avoid long-term sick leave. I’ve had 29 new patients recently, and of them, 13 lived in relationships with violence!* (Rehabilitation coordinator)

#### Violence as conflicts within the family

The explanation that violence often stems from conflicts within the family was given in the interviews. They talked about bad conflict management and that it can occur in any family, but mostly among those who are not trained in managing emotions and therefore conflicts arise. In this regard, the interviewees emphasised that women can also be perpetrators of violence, towards their partners, but also towards children in the family. The notions that violence can be perpetrated by anybody, and that it may occur anywhere, were represented in this sub-theme. The suggested interventions were grounded in therapeutic orientations. This implies that they focused on interpersonal and psychological circumstances, where psychological counselling, conflict management and psychotherapy can be used. One interviewee talked about so-called ‘traditional gender roles’ and how disagreements within a relationship may cause violence in the family:

*Well, I don’t really know, but there’s some kind of stress and they feel tied up and frustrated in the family, and then we also have this issue with gender roles…some just tune in very smoothly, it works well with the children and how to divide the household chores. But sometimes there will be fights about this, women feel constrained and men feel offended…these traditional woman-men patterns, very locked up…and then, maybe, they’ll start fighting more.* (Midwife)

#### Cultural and racialised expressions of VAW

This sub-theme stems from perceptions that violence is more prominent among immigrant people, with a non-Swedish background, who embrace specific cultural norms that support VAW. Interviewees argued that it has to do with how they are raised from childhood and how men and boys in these cultures embody violent behaviours towards women. One interviewee, who worked in a socially and economically deprived area in a large city, described VAW as a sign of cultural differences and religious norms:

*I haven’t always made the connection to violence, but I do work a lot with women who don’t work, who are very dependent on their husbands and, due to religious reasons, are not free to move around by themselves…One must see it as a form of psychological violence. The women are controlled and isolated, and there are many cultural differences here. So, it becomes very difficult for me to ask about violence, because for them* [immigrant women] *it’s so self-evident that they can’t do what they want and can’t be out by themselves in certain places…it’s so self-evident to them.* (Physiotherapist)

#### Violence as unequal gendered power relations

The fifth sub-theme deals with violence as an expression of unequal gendered power relations. It was claimed that the foundation of VAW is related to men’s domination over women in a structure that mirrors society at large, with its gendered power relations between women and men. The concept of ‘machismo’ was mentioned and the dangerous behaviour it fosters among boys and men, with male dominance and control over women, regardless of cultural background. Interviewees gave vivid descriptions of how they had encountered female patients living under terrible circumstances with a controlling and abusive man.

*It’s my conviction that this is about the male role, that it deals with how boys are brought up. I think it’s a gender issue*. (Rehabilitation coordinator)

### Theme 2. The tricky question of asking about violence

The second theme is labelled ‘The tricky question of asking about violence’. Its two sub-themes portray differing views on asking about violence. Firstly, asking about violence is only another work task in primary care alongside all other duties. Secondly, a diversity of obstacles to asking about violence, and justifications for not asking, were highlighted. Thus, everyday practices seemed to differ substantially.

#### Routinely asking about violence

For some of the interviewees, asking about violence seemed to be rather simple and not very complicated. They did not regard it as a very sensitive issue, but instead just one of all the obligations imposed upon them, things that primary care professionals have to do in their clinical practice. Asking about violence was regarded as a way to help care-seekers as much as possible. These interviewees claimed that one should ask everyone, that it should not be a big deal, and that it is equivalent to asking about tobacco use or alcohol consumption, which they always do. Such straightforward actions will reduce the stigmatisation of women exposed to violence, they claimed.

*You know, it’s to play it down somewhat. Compare the issue of asking about alcohol consumption, something that we’ve become increasingly comfortable with. That wasn’t very easy at the beginning either. But one realises that asking about violence is a tool that we have to actually help the patient.* (Physician/manager)

Interviewees commonly highlighted target groups as relevant for routinely asking about violence, for instance pregnant women. But it was also claimed that anyone can be exposed to violence, and that if they observed any signs that did not correspond to the explanations given by the patient, they assumed ongoing violence. In addition, patients with mental disorders, on long-term sick-leave and those with pain problems were regarded as risk groups for exposure to violence.

#### Obstacles to asking

However, asking everyone (as described above) is sometimes difficult. It was claimed that it is a very delicate issue, and not for just anyone to deal with. In order to handle VAW properly, one needs to be trained, it was argued; therefore, inexperience was regarded an obstacle. It was articulated that violence is a very difficult and almost frightening subject for many health professionals to deal with, and therefore they do not dare to raise the question. One interviewee declared that she had been committed to the subject of violence for many years, and she regarded herself as an expert on VAW. Unfortunately, she foresaw that nobody at the clinic would be able, or dare, to deal with violence when she retired:

*Because it…means that you can’t be afraid yourself…*[when I retire] *it will fall apart, I’m convinced, nobody will dare to…but this is the way I work, this is a successful concept.* (RN, Rehabilitation coordinator)

Also, perceptions came to the fore, of not always asking about violence, i.e. when it does not seem to be relevant. Is was argued to use ‘common sense’ and to ‘not ask if a patient has a runny nose’, and a need for selection based on the patient’s reason for seeking help from primary care.

*The question is, kind of, how much are you supposed to go into it…for some patients it could feel quite awkward…so you still need to sift out what seems to be relevant.* (Physiotherapist)

Many patients also come back to primary care on a regular basis, so the interviewees asked themselves how often they should ask the same patient about violence. Once a year? Twice? At every visit? The fact that many patients in primary care are old, and sometimes suffer from dementia, was raised as another difficulty in terms of asking about violence. They asked for more detailed guidelines and policy in relation to this.

Work overload and time constraints were frequently cited as an obstacle to asking properly about violence. Language barriers were another obstacle. It was argued to be problematic to ask sensitive questions in front of an interpreter, and some patients even refuse to have one. Moreover, in an immigrant couple, the husband is often present during his wife’s visit to the clinic and he often speaks Swedish better than she does. Hence, he may steer and control the dialogue with the health professional, which will prevent her from disclosing violence. Also, the risk of being portrayed a racist, or xenophobic was discussed, i.e. when questioning cultural norms among immigrants that may foster VAW. One midwife did not routinely ask pregnant women about violence, because she found it difficult to ask the woman to come on her own, without the husband. Instead, she prioritised including the man in the pregnancy process and did not want to scare him off from being engaged in that.

*No, I don’t ask the woman to come on her own during the pregnancy. I think we talked about this on a course sometime…you know…and there was some disagreement about how to do it…I think it’s so very difficult to exclude the man, and as we want men to also engage in the pregnancy, it feels very awkward to say to the woman like…next time, I want only you to come here. It’s almost like ignoring him, because some men always want to be present when the woman comes to see me, so I think it’s difficult to know if it’s correct to only ask the woman to come. I guess I should notice if there’s something wrong in the relationship…but, yet…maybe one doesn’t do that…?* (Midwife)

Another important obstacle to asking was not being up to date or knowledgeable about the treatment and prevention of violence. There was uncertainty about how to proceed if they receive a positive answer from a patient about being exposed to violence. They experienced uncertainty about available resources and how to make referrals to existing support organisations and networks. They did not always know about the health services’ online checklists, guidelines, available (online) services, web-based educational material and support lines for victims in several languages.

*Well, that centre* [against violence]*…I’ve heard about it, but not of any web-based education. We used to receive emails from the management, but this isn’t something I’ve heard about.* (Physician)

All of this created uncertainty about how to address violence in everyday clinical practice, and the interviewees acknowledged that the issue may become lost in their often very stressful schedules.

### Theme 3. Multiple suggestions for improving primary care work with violence against women

The third theme concerns how the interviewees regarded what has to be done in order for primary care to improve its work with VAW. They asked for more support from managers and they wanted the healthcare organisation to be determined and straightforward in terms of what needs to be done. They also highlighted the difficulties and their concerns about safety arrangements for the record system at the primary care clinic. They claimed that it needs to be updated and developed and they asked for greater competence in the ways in which the medical record system is organised and arranged.

#### Guidelines, expertise and continuing education

The interviewees asked for more detailed routines and guidelines to be disseminated to all staff, not only those who already have an interest in the violence issue. There was consensus among the interviewees that education about violence is essential in order for them to improve and work better with violence at the primary care centres. Everybody must be offered education and increase their competence in how to handle and treat patients exposed to violence, they claimed. At some clinics, interviewees suggested that one person should be sent for education and thereafter inform and teach the rest of the work group. At other clinics, we noticed that there were one or two members of staff who worked intensively and were very engaged with violence and had thereby developed high levels of competence. Again, others wanted everybody to have the same information, so that they could collaborate and develop collective actions.

*It would have been very convenient to have something explicit, the way we always ask about smoking and tobacco and alcohol, that you can have it as a routine question. Because then, yes, partly it should always be included in the conversation and if someone actually asks the question, the patient would probably get the sense that one dares to talk about it a bit more.* (Physiotherapist)

They also expressed a desire for more, and continuing, education on VAW and how to deal with it on a regular basis.

*One can never consider oneself fully educated, it has to be a systematic issue, so it’s very good to have workshops, because then I can be very specific and ask questions, and be reminded of things related to violence.* (Midwife)

*Everybody at this primary care clinic must participate in web-based education on violence. But apart from that …It wasn’t included in my professional training as a physiotherapist, I don’t remember us ever talking about that. But it would certainly be very good to include it.* (Physiotherapist)

#### Organisational determination

The interviewees asked for more resources and time to be allocated to continuing education and professional competence about violence. Furthermore, the work organisation should make information available about contacts with institutions and agencies that specialise in VAW. In addition to all the information that can be retrieved from the internet, they also asked for more discussions and workshops on VAW. Some claimed that violence should be a standing item on the agenda for their regular workplace meetings. Others suggested that one person in the work group should have the specific assignment to be responsible for the issue of violence. In addition, the organisation should work to develop close collaboration with other actors in the field of VAW, such as social services, women’s helplines and women’s shelters. One interviewee expressed concern about not being supported by the healthcare organisation:

*It seems as though there are no regional guidelines that work in practice, so I think there was an initiative from a single person on the staff to develop such guidelines. I think this is a deficiency, not being supported at the regional level.* (Psychologist)

As described above, we observed solitary, well-informed and deeply engaged professionals, (in Swedish *eldsjäl*), who make personal efforts to develop guidelines, arrange internal education for staff and always highlight VAW as an essential question at the clinic. But there was doubt as to the continuation of this work within the organisation:

*When I quit…everything that I’ve built and developed will just fall apart. Nobody will dare continue. If they just understood what I do and if I only had the chance to declare what I do…that this way is successful! Because it’s important that others here at the primary care clinic understand that this is the correct way to work with violence.* (Rehabilitation coordinator)

#### Managerial support

The importance of firm support from managers was one essential demand from the health professionals. The manager was regarded a key person if the workplace is to develop and improve its competence around VAW. If the manager does not prioritise this work, it will fall short and will not become part of everyday practice in primary care. The manager was supposed to set the agenda on VAW for the whole workplace. Prioritising VAW will increase the visibility of the question and professionals’ engagement with it.

*Well, if they want people to participate in educational programmes to deal with violence…well, that’s up to the manager at the clinic to implement. If the managers really consider it a highly important question, and they judge it worthy of investing time and money, well, then they’re good managers with a focus on improvement. Then to really make it happen, to convince people here to invest time in such education, that’s also for the manager to deal with…* (Manager).

*Well, I mean…a wise manager would say ‘let’s put effort into learning more about violence, in the long run, we’ll gain both time and resources.’ I mean…it should be that way.* (District nurse)

## Discussion

Many of the findings of this interview study are in line with several other studies on health professionals’ readiness to deal with VAW, both in terms of obstacles and facilitating factors [7–14]. Similarly to previous research, the findings of this study highlight major concerns regarding Swedish primary care’s readiness and ability to encounter and support victims of violence. Firstly, it seems as though the management and organisation of primary care have failed to disseminate action plans or guidelines for VAW to their employees. Secondly, the professional work on VAW is problematic. Knowledge and awareness about VAW among the professionals we interviewed varied along a continuum, from a non-reflective notion to a fully developed understanding of the mechanisms behind such violence. Thirdly, a novel finding compared to previous research, is the very naming and framing of violence, i.e. the conceptualisation of violence, which varied and was vague. All of these factors may influence interventions and patients’ access to competence and high-quality primary care regarding VAW. We will in the following discuss structural factors such as work organisation, leadership and policy, but also intermediate factors such as professionals’ praxis, knowledge, attitudes and training concerning VAW. Thus, we see three aspects that are vital for the implementation of good care for victims of violence: (a) *organisation, policy and leadership*, (mainly linked to theme 3); (b) *professional work and competence on VAW* (linked to all the three themes); and (c) *the naming of violence against women* (mainly linked to theme 1).

### Organisation, policy and leadership

It was highlighted in the interviews that the regions in Sweden, which are responsible for administrating healthcare, must develop better guidelines and support systems for professionals in relation to VAW. Many of the professionals did not know how to ask about exposure to violence, or what to do if they received a positive answer from patients. We agree with Goicolea et al. [37] that, in primary care, it is important that VAW is prioritised within the work organisation. Furthermore, we argue that there is a need for stronger and clearer national governance. This would help not just the professionals, but also the regions, to work more efficiently with VAW. Currently, the 21 regions seem to be being forced to “re-invent the wheel” over and over again in their work with guidelines and checklists, with the undesirable result that guidelines may look quite different in different regions. In an earlier study, we found that many regions have indeed developed guidelines and checklists for how to handle VAW [19]. However, the implementation of these guidelines seems to be inadequate and awareness of their existence was low in the data material gathered in the current interview study. Furthermore, we argue that it is essential that resources are made available, not least in terms of time allocation. The work stress and lack of time for knowledge development on VAW was one of the major obstacles emphasised in this study.

In order for healthcare organisations to participate in achieving the national, and political, goal that ‘men’s VAW shall come to an end by the year 2026’ [38], they need to address the violence issue in a clearer manner. In its 2016 handbook on violence in close relationships [29], the Swedish National Board for Health and Welfare stresses that new forms and tools are required to ensure closer collaboration between healthcare authorities and other actors in society. This would help to improve healthcare readiness to manage VAW in prevention and treatment interventions.

### Professional work and competence on violence against women

Primary care is an important arena for women exposed to violence, because it is the first health-seeking port of call, and a visit to primary care is needed before further referrals can be made to specialised care within the Swedish healthcare system. However, women exposed to violence often hesitate to spontaneously disclose their experiences or life situation, and they seldom seek help for the violence itself, but rather for different health consequences of the violence, e.g. headaches, stomach-ache, widespread unexplained chronic pain or gynaecological complaints [39]. It is therefore vital that professionals in primary care have the necessary knowledge and skills and a readiness to deal with VAW as a health matter [8, 12]. Sundborg et al., [40] evaluated an educational intervention for Swedish district nurses in primary care and their preparedness to encounter women exposed to violence. They found that the intervention had a low impact on the district nurses’ preparedness. Instead, the authors emphasise the need for persistent and continuing support and supervision, in order to improve readiness. Professionals need to know how to ask about violence and what to do if they receive a positive answer to their questions. This reasoning is supported by the findings of this study. Although we did not aim at comparisons between the professions, we noticed that the interviewed midwives were more aware of violence than other interviewees, especially sexual violence. This awareness is probably a result of the explicit recommendations for midwives in antenatal care by the National Board of Health and Welfare [31, 32], but also because VAW is probably better incorporated into the curriculum of midwives. Accordingly, midwives have for a long time been obliged to routinely ask all women about violence, and not just when there is a suspicion.

The interviewees demanded more educational efforts from healthcare policymakers, and increased support from managers and their work organisation. The importance of training in relation to encountering and treating victims of violence has also been emphasised in several other studies (e.g. [12, 26]). Interviewees in this study highlighted the lack of education and knowledge about VAW. We interpret this as a form of professional ambition in line with the ways in which health professionals have been trained into their professional duties, i.e. working in accordance with evidence-based practice (EBP) [41, 42]. Health professionals are regulated by Swedish law to be up to date with the latest, and best available, evidence and knowledge in order to ensure good clinical practice and well-informed, professional judgements [43]. This implies the need for clear clinical guidelines. It is important to emphasise here that the responsibility for developing such guidelines lies at the national and regional levels, not with the individual professionals themselves. In her critique of the ways in which VAW is understood and dealt with in healthcare, Tower [44] argues that it is problematic that VAW is framed within the medical model of care, meaning that it avoids factors of power dimensions in healthcare and takes a simplistic, and often medicalised, approach to the issue of men’s VAW.

However, we also interviewed professionals who were knowledgeable and up to date on men’s VAW. They had themselves achieved expertise despite a lack of support from their organisation. They were engaged and enthusiastic, working quite independently within their work organisation. In an ideal situation, such professionals may do an excellent job of disseminating their expertise on violence to colleagues. However, there is a risk that the whole issue of how to address violence will fall apart if these driving spirits leave their jobs and the organisation. Therefore, it is vital that, in primary care, the response to violence is integrated into routines and work processes throughout the entire work organisation and that protocols, guidelines and policies are disseminated and followed up. First-level managers are key actors in such implementation. As the interviewees argued in this study, without their managers’ recognition that this is an important work task for primary care, it will probably not become a prioritised topic.

The time constraints that the professionals experienced made them hesitant about dealing with men’s VAW, among all their other duties. During the last three decades, the publicly funded Swedish healthcare sector has undergone an organisational transformation in line with a new public management approach, with an organisational focus on efficiency, time allocation and the marketisation of the public sector, with the specific aim of reducing public spending on healthcare and other welfare services [45, 46]. Because the majority of Swedish healthcare is financed by taxation, this means that new public management affects most healthcare employees, and the organisations now have limited economic resources and a shortage of human capital. We noticed that many of the interviewees focused on the treatment of ill-health (tertiary prevention), rather than health promotion (primary prevention) or disease prevention (secondary prevention). This may be an effect of new public management creating a tight and slim organisation, which hinders them from engaging in new, and complicated, health matters such as VAW. Hence, professionals in primary care have little time, power or opportunity to engage in professional knowledge development; for instance, by searching the scientific literature, or allocating time to engage in continuing education on VAW.

The issue of asking about violence is an example of a new work task that creates problems, frustration and doubts. As presented in our results, it could be regarded simple to ask every woman about violence, no more difficult than asking about diet, alcohol, smoking etc. This is in line with several other studies [47–49]. However, the requirements of asking might also create substantial obstacles and insecurity. Iversen et al. [50] suggest that a way to avoid these problems is to provide team-based collaboration, peer support and counselling, as well as sufficient resources.

Swedish national healthcare regulations on violence against women are constantly changing in accordance with the development of new knowledge, but also in accordance with political will and determination. Hence, new national guidelines were launched while the analysis of the interview material presented here was being conducted. In this latest version of the recommendations, from 2022, a closer collaboration between healthcare, social services and the police, was announced [51]. The instructions about confidentiality between social authorities have also been changed, making it easier for different authorities to collaborate on specific cases of violence. This will hopefully make it easier for victims of violence to receive support after disclosing violence. Moreover, the new recommendations require all healthcare sectors to routinely ask all of their patients about violence, although they still have the right to decide when it is appropriate to ask, or not. At first glance, these new recommendations seem promising. However, leaving space for flexibility about when to ask might reinforce the ambiguities and the insecurity among staff that we have observed in this study.

### The naming of violence against women

From our analysis of the findings of this study, we conclude that the professionals struggled with how to talk about VAW. It was difficult for them to use the appropriate concepts, probably due to a lack of knowledge, or an unwillingness to pinpoint certain groups. As indicated in Introduction of this article, concepts on violence vary considerably in the research literature [15–17, 21, 52]. Gender neutral concepts [19] such as ‘domestic violence’, ‘family violence’ and ‘intimate partner violence’ often ignore the gendered aspects of violence and simply indicate that violence and/or conflicts exist within a family/relationship [20–22]. Users of these concepts often emphasise psychological factors and recommend therapeutic interventions, e.g. family counselling. Courses in conflict management to improve problem-solving skills between partners may be offered to victims of violence.

In contrast, the concept of ‘men’s violence against women and girls’ [21] highlights a direction for the violence, in which men are identified as perpetrators and women as victims. And the concept of ‘gender-based violence’ [52] addresses the gendered power structure within which women and girls are exposed to men’s violence. Men’s violence against women is within this notion regarded as being rooted in unequal power relations and societal structures where masculine ideals of power and dominance intersect and create destructive behaviour. It is emphasised that such structures are dangerous and ‘toxic’ and constitute a threat to women’s and girls’ security [18]. The UN’s Sustainable Development Goals address this view on gendered violence and they consider it a violation of human rights. [16]. Boyle [15] problematises the theoretical multiplicity of gender and violence. She claims that “this multiplicity is not always recognised, resulting in a flattening of distinctions which can make it difficult to recognise the specifically gendered patterns of violence and experience” [15, p.19].

We regard an awareness of the gendered power relations in society as essential for the understanding of VAW as a health matter. In the new national recommendations on VAW from 2022, the gender-neutral concept of ‘violence in close relationships’ is used. The recommendations are entirely gender-neutral and they avoid explaining or understanding the underlying factors of power structures, by not using the terms ‘women’ and ‘men’, and by not highlighting any direction of the violence. A subsequent handbook, building on these new recommendations, was published in 2023 [53], aiming to be a tool for social services, dental care and healthcare. Although it hints at the fact that violence in close relationships affects women and girls more than men and boys, when addressing health professionals’ obligations, violence is described in gender-neutral terms and the requirement to ask about violence seems quite loose, flexible and optional. The questions arising from this are: What will the results of these new recommendations be? Will there be any improvement for victims of violence? Will there be enough training for professionals to enable them to handle VAW correctly? We hope that the new regulations will be disseminated into primary care work organisations, and that additional training on VAW will be implemented, specifically addressing the problems with conceptualisation and gender neutrality. The closer collaboration between different authorities, and the recommendations to ask all patients, are promising developments. However, we also foresee a risk that it will still be up to each region, or even individual middle managers, to decide upon what actions to take to deal with VAW in primary care practice. Thus, there is an obvious risk that the confusion and vagueness will remain.

This study has some *limitations* that need to be addressed. Firstly, the problem with time constraints among primary care professionals that made managers decline participation, may have led to important aspects of VAW, both positive and negative, not being represented in the material. Did they decline participation because they did not want to reveal poor procedures on VAW? Or were there conflicting perceptions among managers and staff on how they work with VAW? Due to the inductive approach of this study, and due to the fact that we had to adhere to ethical guidelines on how to conduct research in healthcare, we cannot say what we missed out. Despite these drawbacks, we consider the result from the interviews useful in developing new knowledge concerning conceptualisation of violence, and on knowledge and practice on VAW in Swedish primary care. We can conclude that in relation to the research question and the questions in the interview guide, we received answers to all of them. Secondly, as one of the authors (CVC) is not Swedish speaking, the close reading of the interview transcripts was conducted by the other two authors (AÖ, KE). On the other hand, her expertise in the field of VAW was a major contribution to the study. The collaboration between the three authors, all with vast experience of researching VAW in different contexts, facilitated an analysis at a higher level of abstraction, including theoretical frames and conceptualisations of gendered violence. Thus, the triangulation between the three authors in this thematic analysis [54], helped us to scrutinise and refine the interviews in greater depth, which improves the credibility. The transferability of the findings from a qualitative study such as this is analytical and theoretical, meaning that they are not generalisable from a statistical point of view, but may be transferable to other social contexts that are similar from theoretical and analytical perspectives [34].

## Conclusions

The findings point to important policy and practice implications in primary care. There was a consensus among primary care professionals that men’s VAW is an important, but difficult health problem that needs to be recognised in primary care. However, knowledge about VAW varied in terms of how the interviewees framed and explained it. The themes describe differences regarding the professionals’ approaches towards victims of violence, ranging from a very thorough and concerned approach to an approach of ignorance with little engagement. Time constraints and a stressful everyday practice seem to be major obstacles to professional development around VAW. Better support and more resources from the healthcare organisation, clearer leadership and more developed policy on VAW would improve and facilitate their work. All of these factors would improve professionals’ readiness to meet victims of violence.

The results of this study are important for the Swedish regions, i.e. the stakeholders in primary care, and will help them to understand the differences, difficulties and struggles among professionals dealing with VAW in their clinical practice. A more developed conceptualisation of VAW in national policy and regulations is also needed. All of these factors are indispensable for primary care’s work with victims of men’s violence against women.

## Data Availability

The data supporting the findings of this study (individual interview transcripts in Swedish) is not openly available because that would violate the terms of the Swedish Ethical Authority. Data may be obtained from the corresponding author upon reasonable request.
